# Use of CAR T-cell for acute lymphoblastic leukemia (ALL) treatment: a review study

**DOI:** 10.1038/s41417-021-00418-1

**Published:** 2022-01-05

**Authors:** Mohsen Sheykhhasan, Hamed Manoochehri, Paola Dama

**Affiliations:** 1 Research Center for Molecular Medicine, Hamadan University of Medical Sciences, Hamadan, Iran; 2 Department of Mesenchymal Stem Cells, Academic Center for Education, Culture and Research, Qom, Iran; 3 Research Fellow School of Life Sciences, University of Sussex, Brighton, UK

**Keywords:** Biomarkers, Diseases

## Abstract

Acute lymphoblastic leukemia (ALL) is a cancer-specific lymphoid cell. Induction and consolidation chemotherapy alone or in combination with different therapeutic approaches remain the main treatment. Although complete or partial remission of the disease can be achieved, the risk of relapse or refractory leukemia is still high. More effective and safe therapy options are yet unmet needs. In recent years’ new therapeutic approaches have been widely used. Hematopoietic Stem Cell Transplantation (HSCT) presents significant limitations and the outcome of the consolidation treatment is patient dependent. Side effects such as Graft versus Host Disease (GvHD) in allogeneic hematopoietic stem cell transplantation are extremely common, therefore, using alternative methods to address these challenges for treatment seems crucial. In the last decade, T cells genetically engineered with Chimeric Antigen Receptor (CAR) treatment for the ALL are largely studied and represent the new era of strategy. According to the Phase I/II clinical trials, this technology results seem very promising and can be used in the next future as an effective and safe treatment for ALL treatment. In this review different generations, challenges, and clinical studies related to chimeric antigen receptor (CAR) T-cells for ALL treatment are discussed.

## Introduction

Acute lymphoblastic leukemia (ALL) or lymphocytic leukemia, is progressive cancer in children and adults. Lymphoid progenitor cells in the bone marrow, blood, and extra-medullary sites undergo malignant transformation and proliferation. The majority of ALL cases are classified as precursor B-cell type, but the T-cell neoplasm is a rare and extremely aggressive phenotype and is slightly more common in adults than in children [1]. According to the American Cancer Society, the estimated number of new cases in the United States (USA) in 2021 is 5,690 including both children and adults. Among all cases, 80% occurs in children and young adults [2].

The treatment for ALL consists of three phases: remission induction, consolidation, and long-term maintenance, with central nervous system (CNS) prophylaxis given at intervals throughout treatment. The frontline of treatment for induction therapy is the standard chemotherapy. Targeted drug therapy can be used alone or in combination with chemotherapy for all three phases [3].

Induction therapy aims to destroy the leukemia cells (blast). Complete remission is achieved when there is no sign of leukemia cells in the blood and the bone marrow after the treatment and normal hematopoiesis is restored. In the consolidation phase, the treatment is intensified and lasts for several months to reduce the number of leukemia cells still in the body and to avert the relapse of the disease [3, 4].

Several chemo-drugs developed over the past 40 years are combined to prevent the resistance, indeed the 5-year survival rates in children and adolescents up to 19 years was only 10% in the 1960s, but increased to 80–90% at present. Approximately 2–3% of patients are refractory to induction chemotherapy, and 10–15% experience relapse [1, 5, 6]. In contrast, the 5 years survival rate in adult ALL in the United States is 68.6 percent, reports the NCI (National Cancer Institute), and about 40% in Europe. Despite 80–90% of the adult patients’ response to induction chemotherapy, only 30–40% of adult patients will achieve long-term remission [3, 4, 7].

ALL is a highly heterogeneous disease, although cases can be stratified into a favorable, intermediate, and adverse-risk group based on the genetic profile that encompasses recurrent chromosomal aberrations as well as a mutation in the genes involved in hematopoietic proliferation and differentiation. Cytogenetics at the time of diagnosis is the single most important prognostic factor for patients treated upfront with chemotherapy regimens alone, and to make decisions regarding the use of allogeneic hematopoietic stem cell transplantation (HSCT) as consolidation therapy [8–10]. HSCT may provide some major complications such as complexity and graft versus host disease (GVHD) [11].

One of the significant obstacles to the cure of acute leukemia is its propensity to relapse after chemotherapy or hematopoietic stem-cell transplantation (HSCT). As a result, scientists pursued novel efficient treatments free of these issues. Many of the approaches are currently being evaluated for ALL salvage and improvements in the therapy of adult ALL are highly encouraging. Targeted agents, such as Imatinib mesylate, a specific tyrosine kinase inhibitor of BCR-ABL, in children with Philadelphia chromosome-positive ALL, have been shown to improve survival when they are combined with conventional chemotherapy in a consolidation phase [12, 13].

In the past decade, immunotherapies involving endogenous T cells have emerged as a new strategy to treat r/r ALL and avoid chemotherapy resistance [14]. The rationale for immunotherapy in ALL is supported by evidence for immune surveillance in the development of leukemia [15].

Chimeric antigen receptor (CAR) T-cells are genetically modified polyclonal T or natural killer (NK) cells with fusion proteins to guide them toward a given molecule in the tumor cell surface. CAR T-cell components include an extracellular antigen recognition domain of the single-chain Fragment variant (scFv) derived from an antibody, a transmembrane domain, and an intracellular signaling domain. Targeting moiety is presented in native form without the need for additional processing within the groove MHC molecules. So, CAR T-cells identify target tumor cells regardless of a patient’s MHC haplotype.

The use of ligand or peptide to target the CAR-T is an area of development. The roles of monoclonal antibodies, chimeric CAR‐T‐cell therapies, and other novel targeted approaches in adult ALL continue to be defined [3]. Their incorporation into frontline adult ALL therapy, in concomitant or sequential strategies, may increase the cure rates to levels achieved in pediatric ALL and may reduce the need for prolonged intensive and maintenance chemotherapy.

In this review article, we discuss CAR T-cell therapies against ALL, focusing on the target antigens used for CAR design, the difference in CAR generations, CAR T-cell clinical trials and FDA-approved, challenges in CAR T-cell therapies, and the latest overcoming strategies.

### CAR T-cell generations

Adoptive cell therapies (ACTs) have been used to treat cancer for over 30 years. The rationale that led to the chimeric antigen receptor-modified T (CAR T)-cells was to overcome the HLA restriction, the effective presentation of target epitopes, and the lack of a broad TCR gene repertoire [16].

CAR T-cells have been investigated in preclinical and clinical studies. In hematological malignancies, the efficacy in targeting cancer encompasses also the complete and long-lasting durable clinical response observed in late-stage chemotherapy-resistant leukemias and lymphomas. Conversely, in solid tumors treatment, the efficacy is yet unmet, and further study is still needed [17–20].

**Fig. 1:**
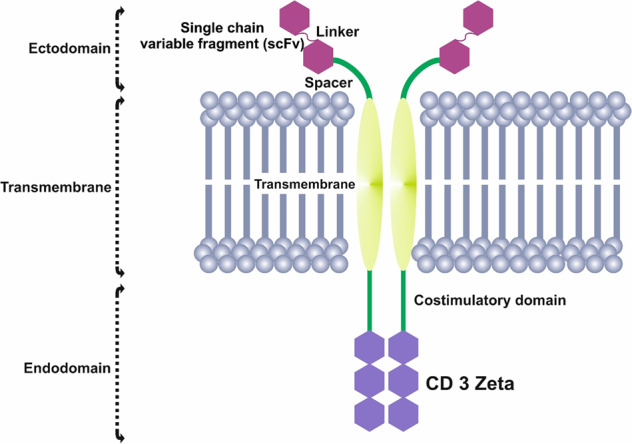
Chimeric antigen receptor CAR structure. A CAR molecule comprises an extracellular MHC-independent antigen-binding ectodomain derived from a monoclonal antibody, including a single-chain variable fragment (scFV), a linker, and a spacer/hinge region, a transmembrane domain, and an intracellular T cell signaling endodomain counting CD3ζ and costimulatory domains.

**Fig. 2:**
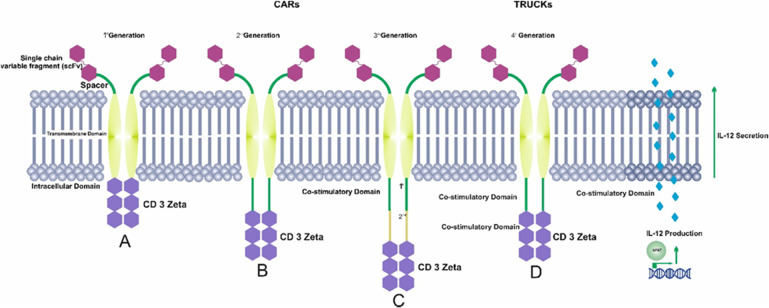
Schematic diagram of four generations of CAR T-cells. CAR T cells can be categorized into four generations, based on domains used in their designs and the CAR structures. (**A**) In the first generation of CARs, there was only one intracellular signal component CD3ζ. (**B** & **C**) The second generation and third generation added costimulatory molecules, one and more than one respectively. (**D**) In the fourth-generation CAR T cells, the recognition of target antigens leads to the induction of cytokine production through the activation of downstream factors.

Chimeric antigen receptors (CARs) have a modular design consisting of an ectodomain, a hinge, a transmembrane domain (TDM), and an intracellular signaling domain. The ectodomain is a signal peptide, an extracellular MHC-independent antigen-binding domain derived from a monoclonal antibody, a single-chain Fragment variant (scFv) formed by the variable portions of heavy and light chains of an immunoglobulin. A spacer lends flexibility and connects the ectodomain to the transmembrane domain (Figs. 1, 2A) [21, 22].

The majority of CAR T cells are designed with immunoglobulin (Ig)-like domain hinges derived from IgG. Long spacers provide extra flexibility to the CAR and allow for better access to membrane-proximal epitopes or complex glycosylated antigens. By contrast, CARs with shorter hinges, including an IgG-derived hinge lacking the CH2-CH3 regions, or hinges derived from native CD28 and CD8 hinges, can be used to target membrane-distal epitopes [23–29].

The transmembrane domain consists of a hydrophobic α-helix that spans the cell membrane mainly derived from CD4, CD8α, or CD28 which confer the stability of the receptor. Further studies have been demonstrated that linking the proximal domain to its corresponding transmembrane domain may enable proper CAR T cell signaling [21, 23]. The intracellular domain of the TCR-CD3 complex transduces the signal in the activation cascade.

According to the structure of the endodomain, based on the number of the costimulatory domain used, CAR T generation passed through four different generations. The first generation used CD3 ζ alone but the administration of cytokines such as interleukin-2 was necessary to increase in vivo tumor rejection. [30, 31].

Afterward, an effort has been invested in understanding the effects of CAR co-stimulation. Additional signals to the engineering T cell can provide the improvement in T-cell effector function and reduce T cell exhaustion.

Studies in mouse tumor models showed that the incorporation of the CD28 or CD137 (4–1BB) signaling domains enhances the antitumor activity and in vivo persistence of chimeric antigen receptors as compared with the inclusion of the CD3 zeta chain alone. These findings are relevant in the context of poorly immunogenic tumors [32]. T cells expressing CARs with CD28/CD3ζ or 4–1BB/CD3ζ signaling domains exhibit differences in effector function, clinical efficacy, and toxicity that are assumed to result from the activation of divergent signaling cascades [33]. Milone et al. demonstrated that CD137 has superior antileukemic efficacy and improved persistence in a primary human acute lymphoblastic leukemia xenograft model and the activity appears to be antigen-independent [34].

In the second generation, an intracellular domain such as CD28 or (CD137) 4–1BB and (CD134) OX40 was added to the cytoplasmic tail of first-generation CARs to overcome the weakening of the T cell proliferation in vitro and its long-term survival after reinfusion (Fig. 2B) [35]. 4–1BB-based CARs have resulted in greater long-term persistence whereas CD28 costimulatory domains resulted in proliferation, survival, and establishment of effector and memory cells and showed faster and higher intensities of phosphorylation, indicating higher signal strengths [27].

CD134 and CD137 elicit T cell proliferation and can play a key role in IL-2 production, survival of T cells, and their perseverance [36].

**Fig. 3:**
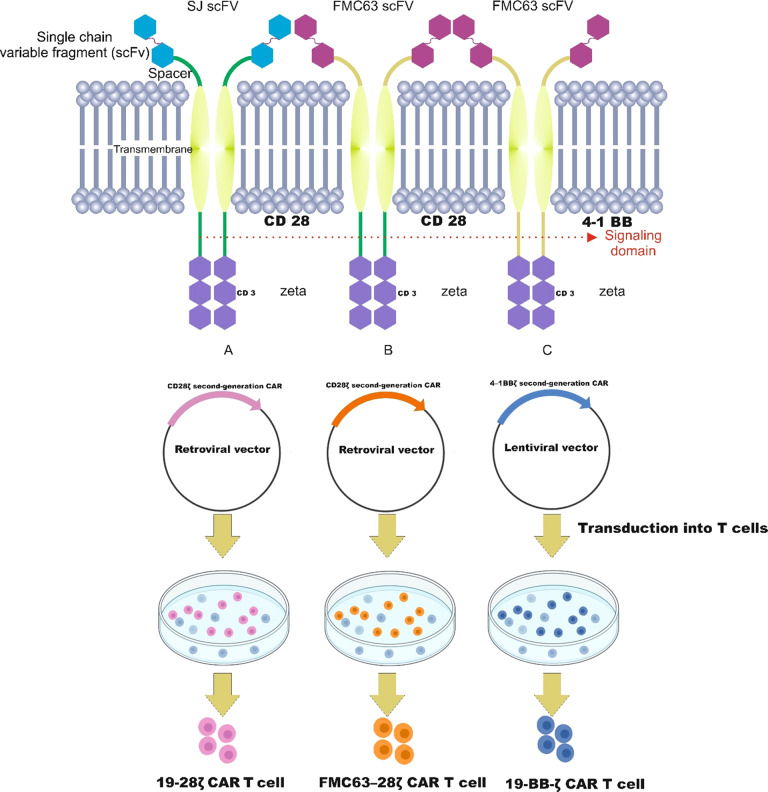
Schematic diagram of different models of chimeric antigen receptors (CARs). **A** At Memorial Sloan KetteringCancer Center and **B** National Cancer Institute, patients have been treated with a CD28ζ second-generation CAR. Respectively, **A** 19–28ζ and **B** FMC63–28ζ constructs were introduced into *T* cells by retroviral transduction. **C** Instead, at the University of Pennsylvania, a 4–1BBζ containing a second-generation CD19-targeted CAR T, was introduced by a lentiviral vector. Moreover, the single-chain variable fragment (scFv) was the difference between the **A** (SJ scFv) group and the **B** and **C** groups (FMC63 scFv).

Most CARs derived from second-generation constructs were used in clinical trials of T cells genetically engineered to express CD19 for patients with B-ALL. Successful second-generation CARs for B-ALL treatment were designed by the University of Pennsylvania (UPenn), the Memorial Sloan Kettering Cancer Center (MSKCC), and National Cancer Institute (NCI) containing scFv, transmembrane, either CD28 or 4–1BB and CD3ζ (Fig. 3) [37–47].

The third generation of CARs was designed to imitate the natural physiology condition and activation of these immune cells by the integration of multiple costimulatory signaling domains, such as CD28, CD137 (4–1BB), ICOS, or CD134 (OX40), into the CD3 zeta domain (Fig. 2C).

At last, CAR T-cells redirected for universal cytokine killing (TRUCKs) are known as the fourth generation of CARs (Fig. 2D). This generation produces IL-12 or IFN-γ and some other cytokines; this strategy could overcome any antigen loss within the cancer cells as a result could induce the immune system to encounter the cancer cells [48]. It is noteworthy that the third and fourth-generation CARs are still in development and not yet approved along with the allogeneic CAR-T [49].

### CAR-T cell manufacturing

**Fig. 4:**
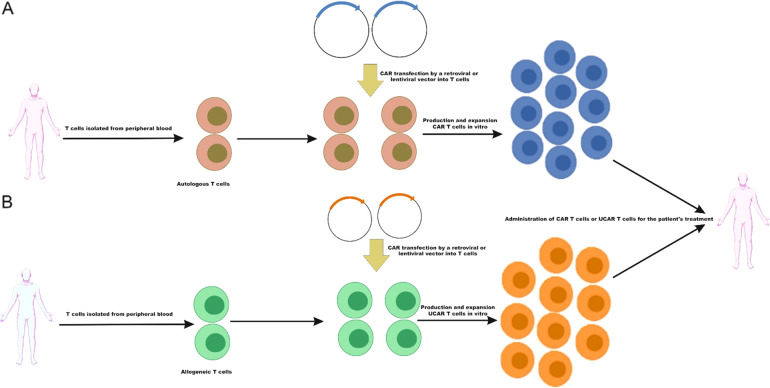
CAR T cell manufacturing and therapy. **A** After extracting autologous T cells from the peripheral blood of the patient, CAR genes are introduced into T cells to manufacture CAR T cells, which are then widely extended in vitro and infused into the patient. **B** After extracting allogeneic T cells from the peripheral blood of a healthy donor, CAR genes are introduced into T cells to manufacture Universal CAR (UCAR) T cells, which are then widely extended in vitro and administered to the patients.

The workflow of the CAR-T cells manufacturing includes isolation of donor T cells, followed by efficient activation, gene transfer of the CAR construct, CAR-T cells expansion, phenotyping, and quality check analysis. (Fig. 4). Leukocytes are taken from patients (autologous) or healthy donor (allogeneic) peripheral blood mononuclear cells (PBMCs). Although most clinical studies use autologous CAR T-cells for B-ALL treatment, the administration of allogeneic CAR T-cells has been also reported in a small and limited numbers clinical study [50]. Several different subsets of leukocytes are used in clinical trials such as CD3 + T cells, central memory cells, naïve cells, and memory stem cells. T cells subsets are separated using specific antibodies followed by the activation process by purifying autologous antigen-presenting cells (APCs) from the patients or donors, or beads coated with anti-CD3/anti-CD28 monoclonal antibodies, or anti-CD3 antibodies alone or in combination with feeder cells and growth factors, such as IL-2.

Lentiviral and retroviral vectors have been widely used in basic research due to the high transfer efficiency and afterward applied for the design and construction of CARs to treat ALL cancers. Because of their safer integration site profile and the ability to infect non-dividing and quiescent cells, lentiviral vectors are more commonly used than γ-retroviral vectors in clinical trials. Lentiviral integration patterns occur far away from the transcriptional sites resulting in a lower risk of mutagenesis [51, 52]. At multiple stages throughout the vector manufacturing process to be used in the clinic, the product is narrowly tested for the presence of replication-competent retrovirus/lentivirus (RCRs/RCLs) to ward off the possibility to be oncogenic as per FDA 2006 guidance. FDA also mandated a long follow-up for RCLs up to 15 years in patients treated for monitoring any potential delayed adverse event using integrating vectors [51, 52].

As we previously described, in the CAR T cell studies, various domains have been used within each of the segments. The antigen-binding domain and the costimulatory domain have been manipulated more than other segments resulting in more variations across the various construct used in the clinic. CAR T-cell therapy constructs against B-cell acute lymphoblastic leukemia include 19–28Z (CD3ζ chain and CD28 co-signaling domain), 19-BBZ (costimulatory 4–1BB and CD3ζ domains and anti-CD19 scFv), TSLPR-BBZ or TSLPR-Z (CD3 zeta intracellular domain, a 4–1BB co-signaling domain, a CD8 TMD, and the scFvs for TSLPR targeting), M971-BBZ or M971–28Z (m971 anti-CD22 monoclonal antibody) [37, 38, 47, 50, 53–61]. Other methods include Sleeping Beauty transposon system and mRNA transfection. The general scheme in clinical trials is similar, however, the methods used are various. The validation requirements are in terms of T cell expansion, T cell transduction, biological activity, quality control testing, and release criteria [21].

Three clinical trials designed to assess the safety and feasibility of CTL019 T cell therapy in relapsed/refractory CD19 + malignancies were conducted at the Children’s Hospital of Philadelphia (CHOP) (Pediatric cohort, Clinicaltrials.gov: NCT01626495) and the Hospital of the University of Pennsylvania (PENN) (Adult cohort, NCT02030847, and NCT01029366).

A phase I/IIa clinical trial performed at the UPenn and children’s hospital of Philadelphia (CHOP) employed a lentiviral engineered autologous T-cell construct expressing a second-generation CAR composed of anti-CD19 scFv, CD3ζ as a signaling domain, and 4–1BB as the costimulatory domain [34]. Indeed, the efficacy in re-inducing remission in patients with multiply relapsed B-ALL is remarkable, demonstrating that the second costimulatory signals have been necessary to achieve relevant T-cell expansion and longer-term persistence in vivo [38, 62, 63].

At Memorial Sloan Kettering Cancer Center [37], and at National Cancer Institute [46] instead, patients received CD28ζ second-generation CAR T cells genetically modified with a replication-defective gammaretroviral vector derived from the Moloney murine leukemia virus. The differences between these cancer centers relied also on the single-chain variable fragment (scFv) as depicted in Fig. 3.

### Target antigens

Several target antigens have been investigated for CAR T-cell therapy in both preclinical and clinical trials. Ideal target antigens for CAR-T cells are homogeneously expressed within tumors, but not on normal tissues, which could cause toxicity by CAR-T cells. Those biomarkers are expressed at different levels on the surface of ALL cells that include the thymic stromal lymphopoietin receptor (TSLPR), CD19, CD22, CD20.

In the case of B-cell malignancies, CD19 expressed throughout B-cell development, was chosen as an acceptable target even if it’s not a tumor-specific antigen, due to its high expression on most malignant B cells, while at the same time lacking expression on hematopoietic stem cells, limiting the risk of aplastic anemia [47].

B-cell aplasia is an on-target/off-tumor effect associated with hypogammaglobulinemia a defect in humoral immunity that can last months to years after treatment, experience recurrent upper and lowers respiratory infections, and result in immunoglobulin G replacement [35, 50, 64–67].

The combination of multi-antigen targeting may increase the effectiveness of this therapeutic approach. The different therapies at the present encompass pooled CAR-T cells, dual CAR-T cells, tandem CAR-T cells, and trivalent CAR T-cells. To prevent tumor antigen escape and to alleviate on-target, off-tumor toxicities, the Boolean logic gates of “AND”, “OR”, and “NOT” have been applied to gate the activity of multi-antigen targeted CAR-T cells. In detail: “AND” logic-gated can be activated in the presence of both antigens simultaneously, “OR” logic gate in the presence of either targeted antigen, finally “NOT” logic gate to avoid the off-target effect [68].

CD20 is expressed on a variety of lymphoid malignancies as a B-cell-specific antigen. CD20-based CAR T-cell cancer therapy has shown high efficacy alone or in combination with CD19 in vitro and in vivo animal models of ALL and CLL [69, 70].

Thymic stromal lymphopoietin (TSLP) is a biological agent similar to IL-7, which could play an important role in hematopoietic cell maturation and pediatric acute lymphoblastic leukemia stimulation [71, 72]. The cognate receptor TSLPR is encoded by cytokine receptor-like factor 2 (CRLF2) gene in a B-ALL cell with a CRLF2 rearrangement. Overexpression of TSLPR is reported in 15% of patients without typical chromosomal rearrangements [73] and is associated with a higher risk for disease relapse. This observation suggests that TSLPR is a novel and attractive candidate for CAR T-cell therapy in some B-ALL cases. Qin et al. indicated that CAR T-cells targeted against TSLPR oncoprotein with 3G11-derived scFv could proficiently eradicate TSLPR expressing cells. TSLPR-based CAR T-cells also have shown therapeutic potential in mice models of B-ALL [74, 75].

CD7 is another transmembrane protein that is typically expressed in T-ALL, not in B-ALL except in rare cases of antigen aberrancy. CD7-based CAR T-cell cancer therapy has been generated and used against malignant T cell lines and primary tumors in a xenograft model of T-AL. Gomes-Silva et al. examined in vitro and in vivo efficacy of CD7-specific CARs for T malignancies. They concluded that CD7-based CAR T-cells have robust antitumor activity in T-cell malignancies [76]. A recent ongoing clinical trial assesses the safety, feasibility, and efficacy of CAR T cell therapy against CD7-positive hematological malignancies in child, adult, and older adult patients [77].

CD38, which is a transmembrane glycoprotein expressed on B- and T-cells, is a candidate therapeutic target for T-cell acute lymphoblastic leukemia whose function is as an adhesion protein related to CD31 or functions as multifunctional ectoenzyme associated with the NAD + and NADP catabolism [78]. Anti-CD38 CAR T-cell demonstrated efficient removal of HTLV-1 + T-cell leukemia [79]. Hofmann et al. recently performed a study for evaluation of efficiency and safety of anti-CD38 CAR T-cells in a 24-year-old female patient with refractory/relapsed B-ALL. They reported a potent and specific antitumor activity of CAR T-38 cells [80]. An ongoing clinical trial is designed for the evaluation of efficiency and side effects of anti-CD38 CAR T-cells in relapsed B- ALL after CD19/CD22 CAR T-cell therapy [81]. In addition, anti-CD38 CAR-T is also being explored in myeloma [78].

### Clinical trials

Clinical trials of CAR T-cells in the treatment of multiple hematologic malignancies, including ALL [38, 39, 46], chronic lymphocytic leukemia (CLL) [16, 82], and non-Hodgkin lymphoma (NHL) [54, 83] has significantly improved the perspective of children with recurrent/resistant disease. The evidence of principle was given in the initial studies of a small group of patients with chemotherapy-resistant disease that showed remarkable responses. The successful treatment of patients via CD19-directed CAR T-cells was the basis for further research on the potential of CAR T-cells targeted immunotherapy of ALL.

A case study has been conducted on two children who experienced chemotherapy-refractory/relapsed in 2013 (ClinicalTrials.gov number, NCT01626495) [16] to answer the question of the capability of chimeric antigen receptor T cells would expand in vivo and have clinical activity. CTL019 as chimeric antigen receptor included a CD137 (4–1BB) signaling domain; infusion of such engineering T-cell was previously reported as promising in the treatment of chronic lymphocytic leukemia (CLL) [55]. Although the cytokine-release syndrome and the development of B-cell aplasia were severe, complete remission was observed in both patients, but one of them had a relapse and loss of the CD19-leukemia expressing cells 2 months later.

Maude et al. extended the investigation to better understand the rate of complete and durability of remission, long-term persistence of chimeric antigen receptor-modified T cells CTL019, to a large cohort of a total of 30 children and adults (ClinicalTrials.gov number NCT01029366). Two blinatumomab-resistance patients and 15 patients who had undergone allogeneic hematopoietic cell transplantation were evaluated in such phase I/IIA Study. A durable remission was achieved in 24 months with overall survival (OS) rate of 78% (95% CI, 65 to 95) and a 6-month event-free survival (EFS) rate of 68% (95% CI, 50 to 92) [38].

At Memorial Sloan Kettering Cancer Center (MSKCC) early studies were conducted by Sadeilan’s group in 2011 [84]. 10 adult patients with chemotherapy-refractory chronic lymphocytic leukemia (CLL) or relapsed B-cell acute lymphoblastic leukemia (ALL) have been enrolled for treatment with autologous T cells modified to express 19–28z, a second-generation chimeric antigen (Ag) receptor specific to the B-cell lineage Ag CD19. The inert CD8 transmembrane domain replaced with the transmembrane and cytoplasmic signaling domains of the T-cell costimulatory CD28 receptor aimed to overcome the lack in most B leukemia cells to express a ligand for activating costimulatory receptors. [Clinicaltrial.gov numbers #NCT00466531 (CLL protocol) and #NCT01044069 (B-ALL protocol)]. The primary point was to assess the safety of infusing 19–28z + T cells, whereas the secondary endpoint was to assess the in vivo function of CAR-modified T cells evaluating the clinical response. The clinical benefit was achieved in the setting of prior cyclophosphamide-conditioning chemotherapy and low tumor burden or minimal residual disease. In the follow-up studies [51] they report the dramatic ability of autologous 19–28z CAR-modified T cells to induce MRD − CRs in five patients with relapsed and/or chemotherapy-refractory B-ALL. Those studies are a breakthrough in the efficacy of the CAR-T therapy approach in patients with an aggressive disease that allowed the transition to a standard-of-care allogeneic hematopoietic stem cells transplant (allo-SCT). In adults relapsed B-ALL has a markedly poor prognosis with an expected median survival of fewer than 6 months. A Phase I protocol in 2014 (ClinicalTrials.gov #NCT01044069) states the complete remission (CR) of 88% in an additional cohort of 16 adult patients with relapsed/refractory B-ALL [37].

In a phase I study by Lee et al. at the Pediatric Oncology Branch of the National Cancer Institute (NCI) at the Clinical Center of the US National Institutes of Health on 20 children and young adults with relapsed/refractory B-ALL a CR of 70% was achieved with the TCR ζ (zeta)/CD28 CAR T [ClinicalTrials.gov, number NCT01593696] [46]. The strength of the study is to provide an accurate response rate in a homogenously treated patient population with a standardized treatment protocol as the authors remarked the differences with the previous report of Davila and colleagues [37].

According to the results and former studies, they corroborate that long-term persistence is not necessary to induce antitumor effects and shorter persistence could have potential benefits because patients treated with this approach do not have severe prolonged B-cell aplasia. Additionally, they showed the correlation of CAR T-cell expansion with both anti-leukemia efficacy and cytokine release syndrome severity and therefore the development of neurotoxicity because of CSF penetration of CAR-T cells.

Park et al. (2018) (ClinicalTrials.gov #NCT01044069) reported data from long-term follow-up in early 2018. CR of 83% with the utilization of 19–28z CAR T cells on 53 adults patients with ALL at a median follow-up of 29 months and a median OS of 12.9 months [85].

Interestingly, this group at the MSKCC found that the best predictor of short-term response and toxic effects was the peak CAR T-cell expansion. In contrast, in the long-term outcomes, the disease burden at the time of the treatment impacts significantly the long event-free survival and overall survival. In this case, the authors didn’t find a significant correlation between the persistence of CAR T cells and survival altogether in the subgroups of patients, which indicates that 19–28z CAR T-cell persistence is not requisite for durable remissions. The lack of correlation with T-cell persistence has been demonstrated in preclinical studies where was shown the high effector function and self-limited expansion of CD28-based CARs [56, 57].

The rationale for the dual antigen targeted CAR T cells is due to the common mechanism of target antigen loss and/or mutation underlying the relapse. Dual-targeted CAR T cells can be generated either with bicistronic CARs that express two different targets ScFv simultaneously in every cell or with mono-CARs.

In a Phase I/II clinical trial, bispecific CAR T-cell therapy called AUTO3 designed to target CD19 and CD22 simultaneously was considered (R/R) B-cell ALL pediatric patients, [58]. OX40 costimulatory domain for the CD19 and a 4–1BB for the CD22 were incorporated for the second-generation CARs. The end-point of the study was the safety and efficacy evaluations. Three dose levels were explored (1 × 106, 3 × 106, and 5 × 106 transduced CAR + T cells/kg) and CAR T cells were infused as a single (for <25% blasts) or split (for >25% blasts) dose based on leukemia burden. 4/4 of patients achieved MRD complete remission with no antigen-negative escape at this early stage.

Yang et al. (2019) enrolled for a phase I study (US NIH Clinical#: NCT03825731) 17 patients with relapsed/refractory B-ALL including 4 pts previously treated with CD19 CAR-T cells. Four were adults, 13 pediatrics (age 1–45). CD19 CD22 bispecific scFv contained a 4–1BB costimulatory signal domain. All pts received a conditioning regimen of fludarabine and cyclophosphamide intravenously before a single infusion of CAR-T cells. The primary endpoints were to gauge feasibility and toxicity, and therefore the secondary endpoints included disease response and engraftment/persistence of infused CD19/CD22 dual CAR-T cells. The study showed safety, and clinical efficiency of CD19/CD22 dual CAR T, noteworthy low toxicity with dose-dependent high CR rate was reported. Nobody relapsed with a median observation time of 60 (7–139) days [86].

Gu et al. (2020) at the Institute of Hematology & Blood Diseases Hospital, Chinese Academy of Medical Sciences & Peking Union Medical College supported their preclinical results proposed a search of CD19 CAR T with scFvs capable of binding to different CD19 epitopes as a choice for patients with mutations in CD19. Despite many efforts that are made to enhance the planning of the CAR, loss of the epitope results in non-responding patients and relapse.

The single-arm pilot study reported the security, and clinical efficiency of CD19 CAR T constructed with a replacement anti-CD19 chimeric antigen receptor (HI19α-4–1BB-ζ CAR T, or CNCT19) in treating 20 pediatric and adult patients with R/R B-ALL. A high CR/CRi rate (90%) was reported after a follow-up of 28 days, median overall survival of 12.9 months, and relapse-free survival of 6.93 months (NCT02975687) [87].

As the authors stated and that we are agreed thereupon, so far a long-term follow-up has revealed a significant proportion of patients relapse after treatment, this suggests that more efforts are needed to spot biomarkers. The exhaustion of CAR-T cells and therefore the subsequent inability to maintain the CAR-T cell-killing effect were major underlying factors for relapse. It is not yet clear whether the expansion kinetics of various T cell subsets are related to differential responses observed concerning long-term patient responses to therapy. Moreover, the antitumor capabilities of some T cell subsets, like CD8 + central memory T cells (TCM), remain controversial and have not been subjected to adequate study. They proposed as a potential biomarker the percentage of CD8 + naïve T cells (TN) based on the results that CAR T cells generated from less differentiated T cell subsets exhibited more proliferative potential and antitumor activity than did those derived from differentiated cell subsets.

In ongoing clinical trials, NCT03984968 at the Hospital of Soochow University as consolidation therapy, T cells expressing CD19 antigen (feeding T cell) were constructed, expanded in vitro, and infused back along with CD19 CAR-T cells into patients to continuously stimulate the therapeutic effect and reduce the relapse rate.

Annesley et al. (2019) showed the effectiveness of CAR T cell product in infant patients. Safety and MRD-CR were similar to those of non-infant ALL patients. However, patients’ number during this study was not enough to form a definitive decision in this regard [88].

Tables 1 and 2 show the list of clinical trials assessing CAR T-cell therapy for pediatric and adult ALL patients that are performed in different centers.

**Table 1:** List of completed clinical trials that use chimeric antigen receptor (CAR) T-cells for acute lymphoblastic leukemia (ALL) treatment.

ClinicalTrials. gov identifier and clinical trial phase	Study title	Ages Eligible for study	Number of enrolled participants or patients	Construct/ Vector	CR and OS rate	Adverse event	T cell persistence/ relapse rate	Median follow-up (Months)	Center(s) or company	Reference
(NCT01626495) I/IIA	Autologous T cells transduced with a CD19-directed chimeric antigen receptor (CTL019) lentiviral vector in patients with relapsed or refractory ALL	1–24 years	30 patients	19.BB.z/ Lentiviral	90%, 78%	100% cytokine release syndrome and 27% severe cytokine release syndrome was observed in all patients. Also, 43% total neurologic toxicity was observed in patients	68% at 6 months/ 26%	7	University of Pennsylvania, PA, USA	CT [38],
(NCT01626495) I/IIA	Phase I/IIA study of CART19 cells for patients with chemotherapy-resistant or refractory CD19 + leukemia and lymphoma (Pedi CART19)	5–22 years	39 patients	CTL019/ Lentiviral	Nr	46% cytokine release syndrome was observed in patients	Nr	Nr	University of Pennsylvania, PA, USA	CT [62],
(NCT01593696)I	Anti-CD19 CAR T-cells for children and young adults with B-cell leukemia or lymphoma	Pediatric, AYA (1 year to 30 years)	53 participants (21 patients)	19.28.z/ Retroviral	67%, Nr	76% cytokine release syndrome and 28% severe cytokine release Syndrome were observed in patients	0% at 6 months/ 17%	10	National Cancer Institute (NCI)	CT [46],
(NCT02030847) I (NCT01029366) II	Anti-CD19 attached to TCR and 4–1BB signaling domains in patients with chemotherapy-resistant or refractory acute lymphoblastic leukemia	Adults (20.6 years to 70.4 years)	35 Patients	CTL019/ Lentiviral	69%, 26-63%	90% total cytokine release syndrome and 40% total neurologic toxicity were observed in patients	Nr/ Nr	13	University of Pennsylvania, PA, USA	[63]
(NCT01626495) I	Chimeric antigen receptor-modified T cells for acute lymphoid leukemia	Pediatric (7–10 years)	two patients	lentiviral	Complete remission in both patients, Nr	fever and severe cytokine-release syndrome in both patients	high levels for at least 6 months/Nr	Nr	Children’s Hospital of Philadelphia	[16]
(NCT01029366) I	Chimeric antigen receptor-modified T cells in chronic lymphoid leukemia	Nr	one patient	lentiviral	complete response 3 weeks after treatment, Nr	Grade 3 tumor lysis syndrome	high levels 6 months after the infusions, Nr	10 months (recent follow up)	National Institutes of Health	[55]
(NCT03289455) I/II	Simultaneous targeting of CD19 and CD22: Phase I study of AUTO3, a bicistronic chimeric antigen receptor (CAR) T-cell therapy, in pediatric patients with relapsed/refractory B-cell acute lymphoblastic leukemia (r/r B-ALL): Amelia study	1‒24 years of age	nine patients	bicistronic retroviral vector	complete response (CR) rates of 70‒90%, Nr	Five patients (63%) experienced neurotoxicity: 4 had Gr 1 and 1 patient (13%) had Gr 3 encephalopathy	Nr	4 weeks’ follow up	Nr	[58]
(NCT02975687) I	Efficacy and safety of CD19 CAR T constructed with a new anti-CD19 chimeric antigen receptor in relapsed or refractory acute lymphoblastic leukemia	3–52 years old	20 patients	lentiviral vectors	90% of patients reached complete remission, overall survival was 12.91 months	the CRS was detected in 95% of patients	persistence of CD19 CAR T cells for >180 days, Nr	median follow-up of 10.09 months	Nr	[87]

*CR* complete response, *OS* overall survival, *Nr* not reported, *CT* https://clinicaltrials.gov.

**Table 2:** List of ongoing, or primary/follow-up clinical trials that use chimeric antigen receptor (CAR) T-cells for acute lymphoblastic leukemia (ALL) treatment.

ClinicalTrials. gov identifier and clinical trial phase	Study title	Ages Eligible for study	Number of enrolled participants or Patients	Construct/ Vector	CR and OS rate	Adverse event	T cell persistence/ relapse rate	Median follow-up (Months)	Center(s) or Company	Reference
(NCT02435849) II	A phase II, single-arm, multicenter trial to determine the efficacy and safety of CTL019 in pediatric patients With relapsed and refractory B-cell acute lymphoblastic leukemia	Pediatric, AYA (3–30 years)	75 patients	19.BB.z/Lentviral	81%, 76%	81% minimal residual disease‐negative remission, 77% CRS, 44% sCRS, 40% ICANS and 13% severe ICANS were observed in patients	83% at 6 months/33%	13	Multicenter	CT [53],
(NCT01044069)I	Efficacy and toxicity management of 19–28z CAR T cell therapy in B-cell acute lymphoblastic leukemia	Adult (18 to 74 years)	16 patients	19.28.z/retroviral	88%, Nr	43.75% severe cytokine release syndrome and 75% minimal residual disease‐negative remission were observed in seven patients	Nr at 3 months/Nr	13	Memorial Sloan Kettering Cancer Center	[37]
(NCT01044069)I	Long-term follow-up of CD19 CAR therapy in acute lymphoblastic leukemia	Adult (18 to 74 years)	45 patients	19.28.z/Retroviral	83%, 95%	67% minimal residual disease‐negative remission and 26% severe cytokine release syndrome was observed in fourteen patients, one patient died.	0% at 6 months/ 61%	29	Memorial Sloan Kettering Cancer Center	[85]
(NCT02028455) I/II	Pediatric and young adult leukemia adoptive therapy (PLAT)-02: a phase 1/2 feasibility and safety study of CD19 CAR T cell immunotherapy for CD19 + Leukemia	Pediatric, AYA (1–26 years)	45 patients	19.BB.z/Lentiviral	93%, Nr	90% cytokine release syndrome, 23% severe cytokine release syndrome, 93% minimal residual disease‐negative remission, 49% ICANS and 23% severe ICANS were observed in patients occurrence of lymphoid to myeloid phenotype was observed in patients	~30% at 6 Months/45%	9.6	Seattle Children’s Hospital	CT [40],
(NCT01860937)I	A phase I trial of T-lymphocytes genetically targeted to the B-cell specific antigen CD19 in pediatric and young adult patients with relapsed B-cell acute lymphoblastic leukemia	pediatric, AYA(1–22.5 years)	25 Patients	19–28z/Retroviral	75%,Nr	80% cytokine release syndrome and 16% severe cytokine release syndrome was observed in patients	Nr	Nr	Memorial Sloan Kettering Cancer Center	CT [143],
(NCT01865617) I/II	CAR T-cells in treating patients with relapsed or refractory chronic lymphocytic leukemia, non-Hodgkin lymphoma, or acute lymphoblastic leukemia	Adult (20 years to 47 years)	29 patients	19.BB.z/Lentviral	93%, Nr	23% cytokine release syndrome was observed in patients	Nr/33%	Nr	Fred Hutchinson Cancer Research Center	CT [39],
(NCT02443831)I	Immunotherapy with CD19 CAR T-cells for CD19 + hematological malignancies	up to 24 years	14 patients	MC63/ Lentiviral	86%, 70%	93% cytokine release syndrome was observed in patients	78.5% at 7 months/40–60%	Nr	University College, London, United Kingdom	[163, 164]
(NCT00466531)I	Safety and tolerability of conditioning chemotherapy followed by CD19-targeted CAR T cells for relapsed/refractory CLL	Adults (43–75 years)	20 patients	gammaretroviral 19–28z vector	25% achieved CR, median overall survival was 17.1 months	Cytokine release syndrome in 100%, grade 3 and 4 CRS, and neurological events in 10%	maximal detectable CAR T cell persistence was 21 days in PB	53 months	Juno Therapeutics	[64]
(NCT03825731)I	Anti-CD19/CD22 dual CAR-T therapy for refractory and relapsed B-cell acute lymphoblastic leukemia	4 adults 13 pediatrics (1–45 years)	17 patients	lentivirus	Near to 100% complete remission at initial, Nr	94% had grade 0–1 cytokine release syndrome and 5.88% patients experienced grade 2 CR	Nr, 50% of patients relapse at 1 year	Nr	Nr	[86]
(NCT00466531) I (NCT01044069) I	Safety and persistence of adoptively transferred autologous CD19-targeted T cells in patients with relapsed or chemotherapy-refractory B-cell leukemias	Adults 48–73 years	ten patients	clinical grade PG13-19–28z vector stocks	no objective disease responses	Most patients experiencing rigors, chills, and transient fevers within 24 h	discrete (CLL-3) or no evidence (CLL-1 and CLL-2) of 19–28z + T cells at 1 month, Nr	Nr	Memorial Sloan Kettering Cancer Center	[84]
(NCT02028455) I/II (NCT03330691) I/II	Clinical experience of CAR T cell immunotherapy for relapsed and refractory infant ALL demonstrates the feasibility and favorable responses	14.5–40.1 months	18 patients	lentiviral vectors	93.3% achieved an MRD negative complete remission	Maximum grade of CRS was 3 and occurred in two of 15 evaluable subjects (13%) and neurotoxicity was limited to a maximum grade of 2	Nr	median follow up of 26.9 months	Nr	[21]
NCT03984968 phase 1	Successful application of anti-CD19 CAR-T therapy with IL-6 knocking down to patients with central nervous system B-cell acute lymphocytic leukemia	46–48 years old	three patients	lentiviral vectors	100% of patients reached complete remission, Nr	Only grade 1 CRS manifesting as fever was noted in patient	high levels of ssCART-19s in serum 2 months after infusions, Nr	The median follow-up was 2.5 years	Soochow University	CT

*CR* complete response, *OS* overall survival, *Nr* not reported, CT https://clinicaltrials.gov.*

### CAR-T toxicity

The two major toxicities associated with CAR-T therapy are cytokine release syndrome (CRS) and the Immune effector cell-associated neurotoxicity syndrome (ICANS) in ALL patients compared to other B-cell malignancies, particularly in adults. The long-term efficiency and safety of this promising approach are not yet available and remain a challenge [59, 60, 89]. The foremost common side effects (incidence greater than 20%) were hypogammaglobulinemia, pyrexia, infections-pathogen unspecified, encephalopathy, headache, decreased appetite, bleeding episodes, coagulopathy, hypotension, nausea, tachycardia, diarrhea, vomiting, viral infectious disorders, fatigue, hypoxia, even poor concentration, and delirium. Additionally, two clinical trials (NCT01626495 and NCT02906371) in ALL pediatric patients reported an acute kidney injury (AKI) and electrolyte abnormalities as a serious complication following the CAR-T treatment [37, 38, 47, 50, 53, 58, 61, 84–87, 90, 91]. Cytokine-associated toxicity, also referred to as cytokine release syndrome (CRS), occurs as an immediate consequence of the activation of macrophages related to inflammatory cytokines triggered by large amounts of IFN-γ released by activated T cells [92, 93]. Across the clinical trials presented in this review, CRS has been reported to occur in any grade in the range of 56–100% including severe CRS (grade ≥) for 3–71% sometimes with a fatal outcome [16, 37–39, 47, 54, 83, 92, 94, 95]. Of note, CRS grading systems differ across clinical trials. Since the beginning of the description of several circulating cytokines elevation leading to the overwhelming majority of symptoms, CRS grading was not clear and lack of consensus available among the institutions made the comparisons between products and trials difficult. CRS must be recognized and treated promptly to preserve life-threatening consequences and to don’t impair the efficacy [96]. The driving cytokine underlying CRS is assumed to be IL-6 [97, 98] indeed, immunosuppression using tocilizumab, an anti-IL-6 receptor antibody, with or without corticosteroids, can reverse the syndrome but could limit the efficacy of the immunotherapy [93].

The first effort to realize a far better refinement of the clinical sign based on the Common Terminology Criteria for Adverse Events (CTC AE v4.03) was achieved by sharing the expertize and experiences of a multi-institutional group of pediatric oncologists in the USA in 2014. Their work has been referred to Lee criteria and has been widely adopted [92]. The algorithm takes patients' response to intravenous fluids (IVFs) and vasopressors, oxygen requirement, and organ toxicities under consideration. Although other different criteria systems wont to grade CRS has been followed, a consensus scale has recently been published by the American Society for Transplantation and Cellular Therapy (16).

Identify biomarkers that allow stratifying the high-risk patients before therapy to intervene preventively could potentially reduce the prospect of developing severe CRS (sCRS) after the infusion.

The approach of Davila et al. in 2015 at MSKCC was to identify a set of criteria for the diagnosis of an sCRS based on the presence of fevers start about 24 h after the infusion with 19–28z CAR T cells, the elevation of specific cytokines, and clinical toxicities as hypotension. Commonly elevated serum proteins during CRS are IL-6, TNF-a, IL-10, INF-g, IL-15, IL-2, IL-10, C-reactive protein (CRP), ferritin, and IL-8 (8, 19–21). Elevation of seven cytokines of 39 measured correlated (*r* = 0.43 to 0.88) to pretreatment tumor burden and also to an sCRS in the cohort of 16 adult R/R B-ALL. Application of those criteria allowed the stratification of the patients into different groups: sCRS will likely require therapeutic intervention with corticosteroids or interleukin-6 receptor blockade and nCRS will experience mild symptoms that would require routine management [37]. Additionally, they found the serum C-reactive protein CRP elevation as a reliable biomarker.

Hay et al. in 2017 in a clinical report of a large cohort of 133 adult patients with CD19 + relapsed/refractory B-ALL, CLL, or NHL reported CRS developed in 70% of patients 62.5% with grade 1 to 3 CRS (grade 1, 26%; grade 2, 32%; grade 3, 4.5%), 3.8% with grade 4, and 3.8% with grade 5. Life-threatening CRS mainly occurred during the CAR T-cell dose-escalation phase of their study. As previously demonstrated CAR-T cell dose was related to the risk of severe CRS in patients with a high tumor burden and an early intervention approach won’t impair the efficacy [39, 95]. In their study MCP-1 evaluation was superior to CRP testing, serum Willebrand factor VWF and the serum angiopoietin Ang-2:Ang-1 ratio were higher before starting CAR T-cell immunotherapy in patients who subsequently developed more severe CRS, suggesting that preexisting endothelial activation might be a previously unrecognized risk factor for severe CRS. The authors remarked the thrombocytopenia before lymphodepletion chemotherapy was also associated with subsequent severe CRS.

Macrophage activation syndrome (MAS) or Secondary haemophagocytic lymphohistiocytosis (sHLH) has been recognized as the causative agent of severe immunological disorders characterized by pro-inflammatory cytokine production, immune-induced multiorgan failure, and lymphohistiocytic tissue infiltration. In rare cases, CRS can evolve into fulminant and refractory HLH/MAS-like toxicities resulting in death [99–102]. One among the foremost important challenges of those severe immunological disorders is that the inability to distinguish MAS/ sHLH within the context of CRS. CAR-T-cell therapy-associated TOXicity Working Group (CARTOX) expanding the Lee et al. criteria developed a consistent approach for monitoring, grading, and management of those toxicities [99, 103]. MAS/sHLH observed at the MD Anderson Cancer Center was reporting in ~1% of all patients [103]. The diagnosis has been made at ferritin levels of >10,0000 ng/ml during the CRS phase within the primary 5 days after cell infusion. Additional therapy with etoposide was given as previously reported [104–106]. Shah et al. 2020 applied such criteria in the Phase I Anti-CD22 CAR-T cell dose-escalation trial using anakinra for treatment of HLH-like manifestations [102].

In association with or following CRS, another common toxicity observed after CAR T cell therapy is neurotoxicity, whose consensus grading scale has also been published by The American Society for Transplantation and Cellular Therapy defined as ICANS. Peculiar symptoms that may occur during or more commonly after CRS, (in a rare case before), vary among patients and encompass, delirium, encephalopathy, aphasia, lethargy, difficulty concentrating, agitation, tremor, seizures, and, rarely, cerebral edema [93, 107]. Neurotoxicity is now considered to be treated separately. Macrophage activation, endothelial activation, and the enrichment of pro-inflammatory cytokines in the CNS have all been proposed as potential mechanisms for CAR T cell-related ICANS [59]. Neurologic symptoms might be observed in association with pathological processes including hepatic failure, severe hypertension, eclampsia, infection, electrolyte abnormalities, and immunosuppressive and cytotoxic drug therapies. The pathogenesis of Neurologic Adverse events (AE) is still unknown, in 2017, Gust et al. reported in 133 adults with refractory B-ALL, NHL, or CLL that followed by the endothelial activation the blood-brain barrier might be disrupted allowing high circulating cytokines to access the cerebrospinal fluid. The presence of fever, high serum IL-6, and MCP-1 concentrations within the first 36 h are predictive of severe neurotoxicity, and early intervention is needed [108]. Tocilizumab has no beneficial effect on ICANS and should even worsen it in some cases highlighting the need for the development of preemptive therapies instead of tocilizumab for management of neurotoxicity [109]. Although it has been proposed that siltuximab with a higher affinity for IL-6 might be preferred over tocilizumab, can’t be considered in the frontline therapy approach as it’s not yet FDA-approved for now.

### CAR T-cell FDA-approved

There are currently five FDA-approved CAR T-cell therapies, including Lisocabtagene Maraleucel (Breyanzi), Axicabtagene Ciloleucel (Yescarta), Brexucabtagene Autoleucel (Tecartus) [110], Idecabtagene Vicleucel (Abecma), and Tisagenlecleucel (Kymriah) [111, 112]. Among them, only Tisagenlecleucel (Trade name of Kymriah™) is indicated for the treatment of pediatric and young adults patients with (R/R) B-cell ALL [113]. Tisagenlecleucel, an autologous CD19-targeted CAR T-cell, recently was approved by Food and Drug Administration (FDA), European Union (EU), and Japan as a cell-based therapy product for use in pediatric patients with refractory or relapsed (R/R) B-cell precursor ALL [114–118]. Based on clinical data, 83% of patients have attained a partial or complete response to a single injection of tisagenlecleucel in a short period [89]. Besides, time left after primary treatment without certain complications, and overall survival was higher for tisagenlecleucel treated patients than other cancer treatment approaches [119]. It is essential to understand that the approved CAR-T therapies all target CD19.

### Advantage of CAR T treatment

There are many benefits for CAR T-cell therapies over conventional treatments. CAR T-cell therapy can help patients who’s their cancer recurs after several treatments to achieve complete remissions for years. Some patients live for long periods without their cancer progress. Major advantages of CAR T-cell therapies are the low number of infusions needed, short treatment period, and rapid recovery than traditional treatments. CAR T-cell therapies also benefit from living cells, which can amplify in patients’ body to provide memory for many years. Therefore, existing long-lived CAR T-cells can recognize and kill cancer cells when there is a relapse. Available data shows a complete remission for a long time in relapse/refectory patients after receiving a single infusion of CAR T-cell therapies. In addition to treating local cancers, this treatment modality can also be successful in eradicating metastatic cells [120].

CAR-T cell therapy is a targeted therapy with high specificity which can remove cancer cells expressing the corresponding tumor-associated antigens, unlike the usual adaptive immune cells. So, to a great extent, this treatment method will avoid unnecessary eliminating of healthy normal cells. Interestingly, CAR-T cells can identify cell surface antigens without the help of major histocompatibility complex (MHC) genes expression. So, cancer cells cannot escape from the immune system (T cell immune surveillance) by hiding MHC or surface molecules contributed to antigen processing and presentation [121–123]. It should be noted that CAR-T cells are almost potentially able to detect different forms of potential antigens including lipids, carbohydrates, and proteins [124]. Furthermore, chimeric antigen receptors have a flexible intracellular signaling domain that allows the cell to prevent the downregulation of costimulatory molecules induced directly or indirectly by tumor cells [125].

### Challenges of CAR-T cell therapy

Although the rate of the primary response to CAR T-cell therapy in B-cell malignancies for relapsed or refractory disease is remarkably effective and is related to a CRi/CR (74–90%) in some clinical trials [37, 38, 47, 50, 58, 84–87] still many patients fail to respond or relapse after the initial treatment. A global study has demonstrated that the relapse rate of CD19-targeted CAR-T cell therapy is ~30%.

This is the results of two important factors: short-term sustainment of CAR T-cells in the bodies of patients with CD19 + cells and therefore the absence of surface antigen CD19 (CD19- variants) and/or surface antigen CD20 (CD20- variants) in patients due to the presence of mutations or deletions in the respective the CD19 or CD20 genes.

In patients with acute leukemia, the downregulation or even loss of antigenic epitope on CD19 appears to be a dominant mechanism of tumor escape. Frameshift code insertion, deletion in CD19 exons 2–5 that encode for the extracellular domain or loss of heterozygosity (LOH) results in epitope loss in 10 to 20% of pediatric B-ALL treated with CD19-directed immunotherapy as detected by clinical flow cytometry [53, 126].

The loss of CD19 targeted epitope followed by the CAR T therapy was reported for the first time on B-ALL patients relapsed 2 months after the success of the treatment. Deep sequencing data showed malignant CD19- clones in the bone marrow and peripheral blood at day 23 [16]. The phenomenon can be explained with the induction of the subclonal selection with a different phenotype due to the result of the therapy suppressing and/or eradicating the leukemic clone identified at the time of diagnosis. Multiple mechanisms have been uncovered that are responsible for the antigen loss considering the main impediment of the promising treatment.

Sotillo et al. reported preexisting alternatively spliced at exon 2 lacking the CTL019 epitope binding site as previously believed and acquired mutations in the malignant B cells of relapsed pediatric patients. The authors highlighted the possibility of targeting alternative CD19 ectodomain based on the evidence that the entire protein is not discarded. In following studies aimed to explore the mechanism associated with the CD19-/r B-ALL, in a cohort study of 12 patients with B-ALL, antigen loss was originated from alterations in CD19 exons 2–5 lead to a truncated protein with a nonfunctional or absent transmembrane domain, whereas alternatively spliced variants were found only with low frequency [40]. These findings were not in support of the splicing hypothesis and suggested an alternative targeting or combination CAR approach. Bagashev et al. reported the presence of mutated and misfolded CD19 in the endoplasmic reticulum of the malignant B cells [127]. Another mechanism of antigen loss is the cell lineage switch. The first case regarding cell lineage switch was reported in 2015. A CLL patient with Richter transformation relapsed after CAR T-cell therapy with a CD19- plasmablastic lymphoma [128].

Afterward, another report showed 2 of 7 patients with mixed lineage leukemia (MLL)-rearranged B-ALL and in a case report of a pediatric B-ALL, relapsing with CD19-negative AML after CD19 CAR T-cell therapy [129, 130]. Ruella et al., reported a novel intriguing not previously described mechanism where CAR T-cell on the leukemia surfaces bound in cis to CD19, covering it from recognizing by the CAR [131].

Partial antigen loss is caused by antigen downregulation and is considered as another mechanism of immune escape to the treatment [132–135]. Watanabe et al. using a CD20 CAR have been the first to set the threshold level of antigen molecules per target cells required to induce lytic function. It has been concluded that CAR-T cells can recognize and lyse cells expressing considerably low levels of the target Ag and were activated and expanded upon such stimulation. This finding suggested that CAR-T therapy might show a better effect in the case of only a limited number of target Ags on the tumor cells [133]. Another research stated that a CD30 CAR could specifically kill lymphoma cells while “ignoring” healthy CD30 + cells due to differential expression of antigen molecules on the cell surface [136]. Walker et al. documented that CAR T-cell function is also influenced by CAR numbers per engineered T cells [134]. Recently, this finding was confirmed in relapsed patients with decreased levels of CD22 on the B-ALL cell surfaces [132].

R/R ALL patients who relapsed after allo-HSCT usually have a poor prognosis, and donor lymphocyte infusion (DLI) can only save a few numbers of patients. The efficacy and safety of donor-derived CARs targeted against CD19 for relapsed B-ALL patients after allo-HSCT administration is studied [137]. Also, it has been reported that the CD123 antigen is overexpressed in ALL patients with relapsed disease. Dual-targeted CAR T-cells against CD19/CD123 were an effective manner for diminishing CD19 antigen loss in animal studies [131]. GVHD rate in Donor-derived allogeneic CAR T treatments remains very low because of low-dose cell infusions [89, 138].

To make sure better expansion, persistence, and safety of CAR-T cells, patients usually receive cyclophosphamide and fludarabine before CAR-T infusion to scale back the occurrence of lymphocytes and lower the burden of leukemia [139]. A phase I clinical trial showed that 22 of 53 adult patients with B-ALL (41.5%) received lymphodepleting chemotherapy followed by 19–29z CAR T-cells expanded severe (grade 3–4) neurologic side effects characterized predominantly by aphasia, encephalopathy, depressed consciousness, myoclonus, and seizure [81]. As a result, this modern treatment should be employed along with specialized medical care by a medical group with different expertise and all the required facilities to certify optimal consequences for patients [60]. So, children with relapsed or refractory ALL, which receiving chimeric antigen receptor (CAR) T‐cell therapy, even need Psychosocial care [140]. Additionally, the cost of this treatment is very high for patients; ~282,000 euros per patient [141].

A CD22 CAR T-cell clinical study also reported that CAR T-cell administration could provide a suitable complete remission (CR) or CR with incomplete count recovery (CRi) (80%) evaluated after 30 days in this study. Most patients only experienced mild cytokine-release syndrome and neurotoxicity [142].

It was shown that preconditioning chemotherapy and reducing disease burden positively affect treatment response without any increase in toxicity [143]. Also, it had been demonstrated that Allogeneic HCT might improve event-free survival following CD19 CAR T-cell therapy [144]. Tocilizumab, an IL-6R antagonistic monoclonal antibody, has been extensively wont to reduce CRS [145].

However, even with the considerable CR rate, ~50% of CD19-targeted CAR T-cell therapies relapsed within a year after treatment. These patients are mostly resistant to secondary treatment with CD22 targeted CAR T-cell therapy. In some cases, relapse happened because of loss of the CD19 antigen. However, CD19 antigen expression remains in the relapsed tumor cells of most patients, and the underlying mechanisms are unknown.

It is offered that CAR molecules can lead to host immune responses because they are immunogenic. This immune response can eliminate infused CAR T-cells. In some patients, anti-CAR immune responses are produced by CD8 T cells. Humanized ScFv has higher CAR T-cell persistency and lowers relapse rate. Vaccination also improves CAR T-cell persistency. Furthermore, 4–1BB CAR T-cell clinical trials have described higher persistency compared with CD28. However, a recent study reported better efficacy for CD28 CAR T-cells [139]. So, the choice between 4–1BB and CD28 targeted CAR T-cells remains controversial. We can bring into account target-mediated toxicity, which results when CAR-T cells damage the healthy cells that express the target, as a challenge in the development of CAR T-cell products. Although this problem can resolve via knocking out the targeting antigen in CAR T-cells by using CRISPR/Cas9 as a new gene-editing technology [146].

Another problem for the development of CAR T-cell therapy is an economical challenge. Currently, the costs of CAR T-cell products and their related costs are also an essential concern for health policymakers, especially in developing countries. However, it seems that by advancement in the field of CAR T-cell therapy overtime work, it can be possible to make its production more cost-effective [147].

Access can be considered as another limiting factor in the term of CAR T-cell therapy. FDA-approved drugs or enrollment in clinical trials are two the crucial access patients. So, challenges with patient recruitment, enrollment, and retention can limit patients’ access to CAR T-cell therapy. Relatively high cost, inclusion criteria limitations, and the uncertain time interval between leukapheresis and infusion can be other limiting factors for access [148].

### Strategies to overcome challenges

One clear way to overcome the antigen loss challenge after CAR T-cell therapy is using more than one target. This strategy is implemented using four different methods: (a) Making different cell populations express various CARs and fusing them sequentially using coadministration; (b) Designing bicistronic or tricistronic vector by expressing two or three different CARs on one cell; (c) Engineering T cells with two different CAR vector (cotransduction), or (d) Encoding bispecific tandem CARs on one chimeric protein by a single vector [135, 149–151]. Transplantation of allogeneic stem cells (allo-SCT) is another potent approach to overcome a patient’s relapse. To decrease the antigen loss and relapse rate, dual-targeted (CD19/CD22) and donor-derived CAR T-cells have entered clinical trial phases. Dual-targeted CARs can be produced as bicistronic or mono-CARs. In the first approach, Engineered T cells are known to express both ScFv simultaneously. As per the latter strategy, all T cells may not be able to express both CD19 and CD22 as the cell population has three different CARs of CD19−, CD22−, or CD19/22-targeted T cells. In CD19/22-targeted cells, the ratio of CD19/CD22 CARs may not be the same. Also, mixed or sequential administration of CAR19 and CAR22 T cells could be used for ALL treatments [152]. A new strategy uses one tricistronic transgene vector that simultaneously expresses three CARs (CD19/CD20/CD22 CARs) on a single T-cell [153]. This approach demonstrates that coexpressing CD19/CD20/CD22 CAR T-cell is regarded as an excellent solution for treatment in ALL, due to simultaneous targeting of CD19, CD20, and CD22. For this reason, increasing the rate of CAR T-cell success and decreasing the relapse rate of ALL due to targeting CD19 − helps escape B-ALL while maintaining their upfront efficacy [150].

As mentioned above, the differentiation of MAS/sHLH from CRS is a crucial challenge in treatment with CAR T-cell therapy. Therefore, efforts are underway to expand the implementation of strategies known to treat/prevent this complication. Predictive biomarkers, including Ferritin and cytokine profiling, were applied to distinguish MAS/sHLH from CRS. Another strategy to treat/prevent this complication is the use of steroids and/or anakinra without affecting CAR T cell efficacy [101, 154].

## Checkpoint inhibitors

Programmed death-ligand 1 (PD-L1) and programmed death-ligand 2 (PD-L2) genes expression, due to the inhibition of CAR T-cell antitumor activity (T cell exhaustion), is one of the most critical challenges for effective use of CAR T-cell in solid tumors and hematological malignancies [155, 156]. Utilizing checkpoint blockade in combination with CARs is one of the strategies to overcome this issue. Additionally, it was reported that checkpoint inhibitors administration could provide an effective and safe improvement in CD19-targeted CAR T-cell therapy in relapsed B-cell ALL [157]. This improvement is due to the release of the immune blockade on the T cell, removing the restriction that’s holding it in check and, in turn, following checkpoint inhibitors, which provide a more significant activity in T cells [158].

In a clinical study performed on 13 children (ranging in age from 4 to 17 years) with relapsed or refractory B-cell ALL treated with CD19-directed CAR T-cell therapy, it was observed that PD-1 checkpoint blockade may improve the CAR T-cell persistence; thus amplifying the rate of CAR T-cell success and reducing the relapse rate of B-cell ALL [159]. Furthermore, mutation design on CD28 costimulatory domain of second-generation CAR T-cells is another strategy that not only improved CAR T-cell durability and decreased exhaustion, but also may be reduced expression of programmed cell death protein 1 (PD-1) in B-cell acute lymphoblastic leukemia (B-ALL) mice [160].

Another strategy for improved CAR T-cell function is these cells engineered to co-express other molecular, including costimulatory molecules, checkpoint blockade, and cytokines which are referred to as armored’ CAR-T. The co-express checkpoint blockade and cytokinesis are thus able to improve the antitumor efficacy of the CAR-T cells, stimulating their pro-inflammatory impact due to their effect on tumor-related dendritic cells (DCs), macrophages, tumor-infiltrating lymphocytes (TILs), and natural killer (NK) cells [161, 162].

## Conclusion

Currently, chemotherapy is considered, as the first line to confront ALL. Stem cells are non-specialized cells that are found in many adults and embryonic tissues. Hematopoietic stem cells have multipotent characteristics, reproducibility, and plasticity. They also have high anticancer potential as a promising approach for the treatment of ALL. For this reason, the transplantation of hematopoietic stem cells has been regarded as a secondary line in human ALL therapy. Although chemotherapy and transplantation of hematopoietic stem cells [Allogeneic stem cells transplantation] remain the gold standard for ALL therapy, the significant complications such as graft versus host disease (GvHD) of these approaches are among their limitations. Due to these issues, novel strategies can be used for ALL treatment. With the advent of knowledge of immunotherapy and its associated methods, increased efficacy in the treatment of cancer was created. Especially, CAR T cell technology as an Immunotherapy-based strategy is suggested as an ideal candidate for ALL treatment. So, this technology could provide great potential in the treatment of cancer. Although it has serious challenges such as cytotoxicity, cytokine release syndrome, neurotoxicity, and ICANS. Development in the production of different generations of CAR technology and their combined use in other ways, such as hematopoietic stem cell transplantation, can be used as an efficient method of ALL treatment after failure of chemotherapy methods. This technology can be used in the future as an effective and safe treatment for ALL treatment.

### Future Perspectives

The central portion of current researches and most likely future investigations are focused on the identification of new target antigens and novel combinations of currently available targets. One principal challenge is to select better preclinical studies to recognize potential combinations. Also, exploration of antigen loss mechanisms and identification of overcoming strategies is essential to the research purpose. Overcoming T-cell function inhibitors in the tumor microenvironment can accelerate the development and advancement of CAR T-cell products. Currently, there are ~470 clinical trials in the field of CAR T-cell therapy and possibly thousands of combinations to study.
